# Participatory prioritisation of interventions to improve primary school food environments in Gauteng, South Africa

**DOI:** 10.1186/s12889-023-16101-z

**Published:** 2023-06-29

**Authors:** Agnes Erzse, Safura Abdool Karim, Teurai Rwafa-Ponela, Petronell Kruger, Karen Hofman, Louise Foley, Tolu Oni, Susan Goldstein

**Affiliations:** 1 SAMRC/ Wits Centre for Health Economics and Decision Science, PRICELESS SA, Faculty of Health Sciences, School of Public Health, University of Witwatersrand, Johannesburg, South Africa; 2 University of KwaZulu-Natal, Durban, South Africa; 3 School of Public Health, University of the Western Cape, Cape Town, South Africa; 4 MRC Epidemiology Unit, University of Cambridge, Cambridge, UK

**Keywords:** Food environment, School nutrition, Priority setting, Stakeholder engagement, Behaviour change, South Africa

## Abstract

**Background:**

In South Africa, overweight and obesity affect 17% of children aged 15–18. School food environments play a vital role in children’s health, influencing dietary behaviours and resulting in high obesity rates. Interventions targeting schools can contribute to obesity prevention if evidence-based and context-specific. Evidence suggests that current government strategies are inadequate to ensure healthy school food environments. The aim of this study was to identify priority interventions to improve school food environments in urban South Africa using the Behaviour Change Wheel model.

**Methods:**

A three-phased iterative study design was implemented. First, we identified contextual drivers of unhealthy school food environments through a secondary framework analysis of 26 interviews with primary school staff. Transcripts were deductively coded in MAXQDA software using the Behaviour Change Wheel and the Theoretical Domains Framework. Second, to identify evidence-based interventions, we utilised the NOURISHING framework and matched interventions to identified drivers. Third, interventions were prioritised using a Delphi survey administered to stakeholders (*n* = 38). Consensus for priority interventions was defined as an intervention identified as being 'somewhat' or ‘very' important and feasible with a high level of agreement (quartile deviation ≤ 0.5).

**Results:**

We identified 31 unique contextual drivers that school staff perceived to limit or facilitate a healthy school food environment. Intervention mapping yielded 21 interventions to improve school food environments; seven were considered important and feasible. Of these, the top priority interventions were to: 1) “regulate what kinds of foods can be sold at schools”, 2) “train school staff through workshops and discussions to improve school food environment”, and affix 3) “compulsory, child-friendly warning labels on unhealthy foods”.

**Conclusion:**

Prioritising evidence-based, feasible and important interventions underpinned by behaviour change theories is an important step towards enhanced policy making and resource allocation to tackle South Africa’s childhood obesity epidemic effectively.

**Supplementary Information:**

The online version contains supplementary material available at 10.1186/s12889-023-16101-z.

## Contribution to the literature

- Adapting and implementing global school nutrition policies in low- and middle-income countries is challenging. Local evidence generation is key.
- This study advances our understanding of context-specific factors that influence the healthfulness of primary school food environments and outlines priority actions that key stakeholders perceive as highly important and feasible to implement.
- The study offers a novel method that draws public attention to the benefit of engaging key stakeholders and using behaviour change theories in intervention design and decision-making processes.
- Results can be a powerful starting point for adopting new or enhanced measures to improve  school food environments.

## Background

Childhood overweight and obesity is a major global health challenge. Approximately one-third of the 340 million children aged 5–19 years estimated to be overweight globally in 2016 lived in low- and middle-income countries (LMICs) [1]. In 2021, 17% of children aged 15–18 years in South Africa were overweight or obese [2]. The World Obesity Federation has estimated that 28% of South African children (5–18 years) will be overweight and obese by 2030. Notwithstanding South Africa being less than 1% of the global population, it is in the top 10 of overweight and obese children globally [3]. This is of concern given the significant health and economic consequences of overweight and obesity for South African children, their families and society [4, 5].

The consumption of unhealthy ultra-processed foods from early ages [6, 7] is a significant threat to child health through increasing rates of childhood obesity and overweight with later life consequences. The food environment that shapes norms and values of food consumption, through the ways food is priced, labelled, marketed, and provided, significantly influences children’s health and well-being [8]. For children, who spend extended periods of time in school and consume up to 30% of their daily calories there [9, 10], ensuring a healthy school food environment is critical. School food environments encompass the physical spaces, infrastructure and conditions within and outside of school premises where food is accessed, obtained, purchased and/or consumed (i.e., tuckshops that are endorsed food retailers on school premises, canteens, food vendors, vending machines) [11]. It considers the nutritional content of food, the price, as well as its availability and promotion (i.e., marketing, advertisements, branding, food labelling, packaging) [11]. As a recognition of its significance to public health, improving the quality and healthfulness of food environments was a key focus of the Rome Declaration on Nutrition (2014). This global commitment emphasises the need for a comprehensive, multi-sectoral approach to address the burden of obesity and overweight by ensuring access to healthy, nutritious food, as well as the knowledge and resources for making healthy food choices [12].

Internationally, many jurisdictions have adopted nutrition policies to improve school food environments [13]. Through its National Obesity Strategy (2015–2020), the South African government has committed to supporting obesity reduction and prevention by creating enabling environments with increased availability and accessibility to healthy food choices in various settings, including schools [14]. Nevertheless, school-based policies, including the South African Integrated School Health Policy [15], pay inadequate attention to food environments and their complexity [11]. Currently implemented government initiatives aim to improve children’s food intake through the National School Nutrition Programme – a school feeding programme—and form healthy eating habits through nutrition education in multiple subjects [16]. The types of food that are sold and marketed to children within and around the school premises remains unregulated despite national guidelines in place to establish healthy school tuckshops [17]. As a result, unhealthy foods bought from school tuckshops or vendors continue to be low in nutrients and high in energy, salt, trans-fat and sugar [9]. This impacts 50% of school-going children in South Africa who regularly buy food at school and do not pack lunch boxes [9]. Finally, there is evidence of ongoing unhealthy food marketing in schools, despite food producers’ voluntary pledges to desist from such practices [18].

Gaps between policy and implementation are not unique to South Africa. Implementing and maintaining school nutrition policies within real-world school settings have proven to be a complex process in LMICs and high-income countries alike [19]. As such, three overarching recommendations emerge from the literature in support of developing and implementing effective school food and nutrition policies. First, explicit consideration should be given to contextual factors mediating or moderating policies’ impact [19, 20]. Without a granular understanding of the local context that shapes eating behaviours, policies often designed for high-income settings might be undermined [19]. This is because schools in low- and middle-income settings may face different challenges such as insufficient resources and poor infrastructure, and lack of access to drinking water, toilets, and electricity [21]. Second, engaging with the public and stakeholders at broad system levels could enhance the identification of contextual factors and inform appropriate design of school food policies [19, 22, 23]. Third, strategies should consider theories of behaviour change through which policies are expected to work [22–24].

As South Africa is moving towards universal health coverage through the implementation of a National Health Insurance Fund, prioritisation of services will need to be responsive to local contexts and reflect societal needs and values [25]. Therefore, it is imperative to identify context-specific strategies for overweight and obesity that complement or enhance the potential of existing efforts in creating safe and enabling food environments in schools. As such, the aim of this research was twofold. First, to understand what needs to change to improve the healthfulness of public primary schools’ food environment in Gauteng province, South Africa. Second, to systematically identify and prioritise context-specific and evidence-based interventions with a diverse range of stakeholders from multiple sectors.

## Methods

### Study design

**Fig. 1:**
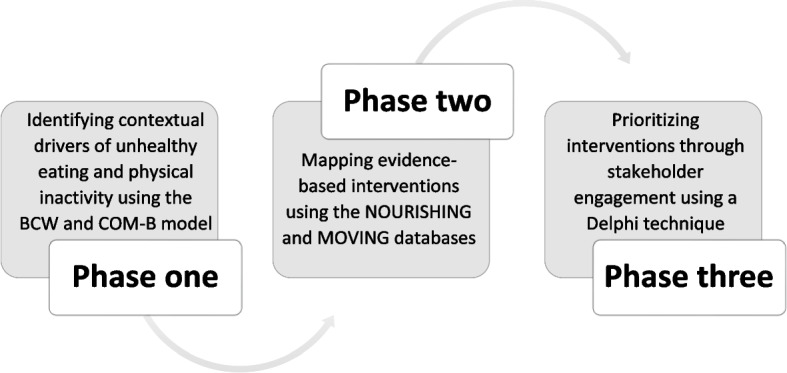
Flowchart outlining the three-phased iterative study design [26]

The research was part of a multi-site study in South Africa (Gauteng province and Cape Town metropolitan) and in Cameroon (Yaoundé) that sought to develop intervention options and inform programmatic priorities in the immediate food and built environments of children and adolescents in and around schools and the home neighbourhood to support healthy eating and physical activity [26]. The study was underpinned by a novel mixed-method intervention design process anchored in participatory methods of stakeholder engagement and behaviour change. In particular, the study drew on the Behaviour Change Wheel by Michie et al. [27] to develop context-specific interventions to improve these environments in three stages: 1) identifying contextual drivers of unhealthy food and built environments, 2) mapping these drivers onto evidence-based interventions and 3) prioritising interventions utilising a Delphi approach (Fig. 1). A separate paper fully describes the methods of the study and the conceptual frameworks [26]. The present paper reports on the application of the three-phased methodology and findings in Gauteng province, South Africa. Gauteng is the hub of the South African economy [28] and was home to about 12% of all South Africa’s schools and 19% (2.56 million) of all students in 2021 [29, 30].

#### Phase 1: Identifying drivers of unhealthy school food environments

In the first phase, we conducted a qualitative secondary analysis (QSA) [31] of data collected for another study [18]. QSA refers to the process of analysing existing data collected as part of previous studies. Instead of collecting new data, researchers leverage available data sources to address new research inquiries or gain additional insights beyond the primary study’s objectives [31]. The present QSA utilised primary data that comprised of 26 semi-structured interviews (that were recorded and transcribed verbatim) with principals, tuckshop managers and school governing body representatives in public primary schools in Gauteng province [18]. The primary study was designed to investigate the availability and advertisement of sugar sweetened beverages in schools after a large beverage entity pledged to no longer sell and advertise their products in primary schools. In addition to the specific focus of the primary study, participants were also asked about existing school policies concerning the food environment, their perceptions of the types of food and beverages should or should not be sold within and around school premises; aspects that fell outside of the scope of analysis in the primary study. To this end, the primary data provided enough material for a secondary analysis to investigate perceived barriers and facilitators and identify opportunities to improve the school food environment using the Capability, Opportunity, Motivation and Behaviour (COM-B) model and the Behaviour Change Wheel [27]. The Behaviour Change Wheel is a visual representation of the COM-B determinants with related intervention and policy areas [27]. Primary study procedures included purposive sampling of interviewees to ensure maximum diversity regarding of schools’ socioeconomic and geographical characteristics, the type of food outlets on premises (tuckshop/informal vendors/vending machines), and the type of food and beverage available in schools [18]. Prior to the interviews, all participants provided written consent that also secured permission for future utilisation of the data for research purposes, including the QSA. QSA was completed after findings from the primary study were published, and it adhered to recommended best practices for conducting this type of analysis [32]. One author (AE) who was involved in the primary study had sufficient first-hand knowledge of the data to ensure scientific rigour in the QSA and appropriate interpretation of the original data.

A framework analysis method was used for the QSA [33]. Analysis began by uploading clean transcripts (*n* = 26) into MAXQDA 2020 (VERBI Software, 2019) [34]. TRP, AE, and PK familiarised themselves with the data and independently coded the first three transcripts to categorise responses relating to facilitators of and barriers to healthy school food environment. Coders met to discuss and resolve any discrepancies in coding and grouped codes with similar content under specific categories, creating a working analytical framework. The framework, categories and codes were then applied to the next five transcripts. TRP and AE met to discuss emerging themes. This process was repeated with subsequent transcripts until no new codes were identified in three consecutive transcripts indicating data saturation. The final framework was used to code all transcripts by TRP and verified by AE, PK and SAK.

Data were charted using a matrix in Microsoft Excel. When populating the matrix, TRP and AE extracted data on barriers and facilitators as they related to the COM-B model: capability (physical or psychological), opportunity (physical or social), and motivation (reflective or automatic). Barriers and facilitators were further elaborated on using the Theoretical Domains Framework (TDF) [35]. TDF is a theory-based model used to analyse behaviour change; it consists of fourteen domains covering behavioural determinants. We mapped these domains onto the COM-B components (see Additional file 1). Assigning TDFs to identified barriers and facilitators allowed us to gain a more granular understanding of factors influencing behaviour which is critical for appropriate and targeted intervention design. Verbatim quotes from participants have been selected to illustrate how school staff’s accounts were linked to contextual drivers. Reporting of the findings adheres to COREQ guidelines [36].

#### Phase 2: Intervention mapping

In phase 2, we identified evidence-based interventions that could address contextual drivers identified in phase 1. PK and SAK conducted a desk-based review of the NOURISHING database [37] that collects policy actions from around the world implemented at a national level and which had evidence of effectiveness in promoting healthy diets at the time of the research. PK and SAK reviewed all sixty-six NOURISHING interventions and selected those most relevant to address facilitators and barriers described in phase 1. Where a NOURISHING intervention was too broad to address a specific driver, the intervention was modified through additional context-specific details to directly address the drivers. Next, interventions were linked to phase 1 contextual drivers and were assigned relevant intervention functions and policy categories. Additional file 1 provides a detailed overview of how evidence-based interventions were adapted and matched with contextual drivers through the application of the Behaviour Change Wheel.

#### Phase 3: Prioritising interventions

In phase 3, the contextual drivers of phase 1 and related phase 2 evidence-based interventions were presented to a stakeholder panel who were asked to prioritise interventions using a “ranking-type” Delphi survey [38]. We purposefully selected stakeholders from four groups whose work and interests were likely to be relevant to strategies that address the unhealthy food environments in schools. The groups included public primary school principals (group 1), school nutrition coordinators and tuckshop managers (group 2), policy makers from the Department of Health and Department of Education (group 3), and academia and civil society (group 4). Following Haynes et al. [39] we aimed to recruit 15 individuals per group for the first survey. Of the 62 individuals who were invited to participate through emails and telephone contacts, 38 agreed to take part. Stakeholders remained the same throughout the entire process, providing continuity and consistency across all rounds to reach consensus. Prior to distributing the first survey, stakeholder groups were invited to a virtual meeting. The research team presented the project goals and processes, the current state of the South African school food environment, its health implications, phase 1 barriers and facilitators to improve these, and the 21 phase 2 interventions. Researchers used clear and simple language to describe the interventions. Plain language descriptions were tested for ease of understanding with individuals who were not familiar with the research topic or methodology. Stakeholders were invited to comment on interventions, an engagement process that resulted in no change to interventions. For stakeholders for whom virtual meetings were not an option, this information was provided before administering the first survey in person. Google Forms was used to develop and distribute each round of surveys. Participants were emailed a link to a consent form and the first survey round, which they were asked to complete within 3 weeks. Regular email reminders (*n* = 3) were sent to those who had not yet completed the survey. When online survey completion was not feasible, a member of the research team took a hard copy of the consent and survey to the stakeholder which was self-administered then captured electronically.

In round 1, stakeholders were advised to read the list of 21 phase 2 interventions and they were invited to separately rate the relative importance and feasibility of implementing each intervention using a 5-point Likert scale. Responses to round 1 were pooled from all stakeholder groups. Following the data analysis method by Latif et al. [40] the collective response median value and the quartile deviation (QD) of each rating were calculated for the whole group and used as a reference for the degree of importance, feasibility, and consensus (Table 1). 

A median score between 4 and 5 was defined as highly feasible and important and a QD < 1 was used to indicate moderate to high consensus. Interventions with the median of 4 and 5 and an QD < 1 were selected for round 2. In round 2, stakeholders had 3 weeks to rate the narrower list of interventions using the same 5-point Likert scale as in round 1. Responses to round 2 were pooled again and only 7 interventions with a median of 4 and 5 and with high level consensus (QD ≤ 0.5) were included for the final round.

Stakeholders in round 3 were asked to rank the interventions from the highest priority (first) to the lowest priority (last) to improve the school food environment. To determine the overall ranking of final round interventions we used the Borda count method [41], an approach to aggregating individual ranked preferences. The ranking was 1 to 7 (number of interventions in round 3). We counted the number of times each intervention was ranked 1 to 7; we multiplied this by the ranking number (1 to 7) and then added this up to determine the total Borda count. The intervention with the highest Borda counts was the one that was ranked the highest.

### Ethical considerations

The study was conducted according to the guidelines laid down in the Declaration of Helsinki and all procedures involving research study participants were approved by the University of the Witwatersrand Human Research Ethics Committee (Medical) (Clearance certificate number M180330) and the University of Cambridge (PRE.2019.105).

## Results

### Phase 1 results: Identifying contextual drivers

The framework analysis using the COM-B and TDF revealed 31 unique contextual drivers that school staff either perceived to limit or facilitate a healthy school food environment. Capability was the least commonly reported component, while factors related to motivation were the most reported. More barriers than facilitators were identified. Most facilitators linked to opportunities within the school’s organisational culture and staff’s motivation to encounter positive change for healthier food environments. Table 2 provides an overview of contextual drives across the COM-B components and TDF domains with illustrative quotes from participants.

**Table 1:** Level of consensus, feasibility, and importance

**Quartile deviation (QD**^a^**)**	**Level of consensus**	**Median**	**Level of importance & feasibility**
QD ≤ 0.5	High	4 and above	High
0.5 < QD < 1.0	Moderate	3 and less	Low
QD ≥ 1.0	Low	-	-

^a^The formula for QD was: Inter quartile range / 2 = Q3—Q1 / 2

### Phase 2 results: Identifying evidence-based contextual interventions

Phase 2 intervention mapping yielded 21 interventions that could improve the school food environment. Interventions cut across all seven policy categories and all nine intervention functions of the Behaviour Change Wheel [42].

Additional file 1 provides a detailed overview of the combined result of phase 1 and 2 and in Table 3, we present the plain language description of the 21 interventions that formed the basis of the first Delphi survey in phase 3.

**Table 2:** Contextual drivers across the COM-B components and TDF domains

COM-B Component		Theoretical Domain Framework	Contextual driver	Illustrative quotes
CAPABILITY	Psychological	Knowledge	• Awareness of the benefits of healthy eating and the harms of sugar ( +) • School staff’s awareness of nationwide efforts against sugar ( +) • Perceived limited memory span of children to retain long-term health knowledge (-) • School staff’s lack of awareness of guidelines on food and beverages (-)	You can explain to the kids why [ultra-processed foods are harmful], we talk about constipation, flavorants, and colorants… at that time they are like WOW. But after a day is like they have forgotten *(Deputy principal, fee-paying school)*
		Behavioural regulation	• Lack of adherence to food and beverage guidelines by children, school tuckshops, and vendors outside of schools (-)	Vendors have the contract with the school governing body. It is for them to stick to what the school wants to be sold […] but they will always sometimes go out of way *(Principal, no-fee school)*
	Physical^a^			
OPPORTUNITY	Physical	Environmental context and resources	• Lack of school infrastructure to promote healthier eating (i.e., taps, covered eating space, fridges, gardens) (-) • Lack of affordable fresh nutritious foods in schools (-) • Cheap and unhealthy foods and beverages at school tuckshop (-) • Influence of product characteristics (i.e., shelf life, pricing) on tuckshop stock (-) • Mismatch of school location, children needing support and National School Nutrition Programme allocation policy (-)	Instead of the acidic drinks we could have fruit juice and it would be good to have some water been sold at the school. Like today, we don’t have water in the taps *(Deputy principal, fee-paying school)* I used to have apples and stuff like that, but they didn’t sell. A child doesn’t want to pay R3.00 [$0.163] for an apple they rather pay R3.00 for an ice-tea *(Tuckshop manager, fee-paying school)* I don’t stock yoghurts and fruit juices because of the sell date. These take a long a time to sell and if the sell by date is gone, I have to take it off the shelve. Then the school has to bear the loss *(Tuckshop manager, fee-paying school)*
		Organisational culture/ climate	• Acknowledged support from donors ( ±) • School’s reward culture using unhealthy foods (-) • Presence of school food and beverage rules and regulations ( +) • Food and beverage monitoring efforts (lunch tins by staff, official health inspections) ( +) • Presence and influence of a school governing body ( +)	Because it is a donation, we don’t have the right to demand anything. They [donors] send fruits, juice, cold drinks, so it depends on what the donation is that those kids get *(School tuckshop manager, fee-paying school)* We sit down with them [vendors] and tell them what we expect them to sell here at school *(School nutrition coordinator, no-fee school)* We have it in our school code of conduct they may not bring any cold drinks / gassy cold drinks to school *(Deputy principal, fee-paying school)*
	Social	Social influences	• Children’s and caregivers’ brand recognition of unhealthy drinks and foods (-) • Social norms for unhealthy eating and inactivity influence on children (peers and households) (-)	There are some kids who really go for the brand name, and they want the real coke *(School tuckshop manager, fee-paying school)* It doesn’t help if you have a policy of “only healthy food” and your children is brought up at home with coke, chocolate, and cake. Then you are banging against a closed door *(School tuckshop manager, fee-paying school)*
MOTIVATION	Automatic	Emotions	• Tuckshops’ fear of loss of profit if stocking healthy vs unhealthy foods (-) • Fear of children getting sick from vendor foods ( ±) • School staff’s fear of negatively influencing livelihoods of vendors (-) • School staff’s and tuckshops’ feeling of pointlessness, discouragement due to lack of collective action among food outlets (-) • Children’s and caregivers’ individual preference for unhealthy over healthy foods (-)	This is a profit-making business and she [tuckshop manager] is going to sell what the children want in order for her to survive, because she pays the school rent. So, she is going to sell the wrong [unhealthy] things *(Principal, fee-paying school)* It is difficult because I can’t be the only one implementing that [healthy options] and everybody around me is not *(School tuckshop manager, fee-paying school)*
	Reflective	Roles and identity	• Convenience and affordability of unhealthy food guides caregivers’ food provisioning, and children’s purchasing (-) • Association of wealth status and unhealthy foods (-) • Stigma associated with National School Nutrition Programme (-) • Caregiver’s perception of school’s sole responsibility to ensure healthy diets for children (-)	What are the quickest things to give children and what children like is chips and sweets and a cold drink. All processed foods but the quickest. The easiest way for parents *(Principal, fee-paying school)* The more take away [junk food] you can, buy it shows wealth, if you got like McDonald *(Deputy principal, fee-paying school)*
		Beliefs (of interviewees) about capability	• Difficulty of implementation of school policies (-) • Difficulty to control vendors outside of school premises (-) • Perceived inability of younger children to control their health (-) • Difficulty of breaking children’s and caregivers’ unhealthy eating habits/mindsets (-)	Policies are brilliant on paper, not always easy to incorporate or to carry out but is important *(Principal, fee-paying school)* Outside the school it’s a challenge. There are people standing on the streets, selling, and we don’t have access to them saying that you must sell this and you mustn’t sell this. It's out of our control *(School nutrition coordinator, no-fee school)*
		Intention (of interviewees)	• School staff’s intent to encourage healthier dietary habits among caregivers and children ( +)	We sell your normal soft drinks and those are actually problematic because they also contain a lot of sugar. That is something we must actually look at and see if we can cut down on that *(Principal, fee-paying school)*

^a^No data has been reported on this component. ( +) indicates a facilitator, (-) indicates a barrier; ( ±) indicates a barrier that can act as a facilitator

**Table 3:** List of evidence-based interventions relevant to address contextual drivers of and improve primary school food environments in Gauteng

1. Government to provide resources to schools, for example taps, vegetable gardens and places for children to eat
2. Recognise, regulate, and allow informal vendors to sell healthy food and drinks on school property
3. Incentivise school tuckshops to sell healthy food and drinks by giving subsidies or decrease tax
4. Introduce peer nutrition programmes, for example school gardens, science days, markets, and other programmes organised by the scholars
5. Train school staff through workshops and discussions to improve nutrition environment within the school
6. Compulsory, child-friendly warning labels on all unhealthy food products
7. Help children make healthier choices by increasing the availability and appeal of healthier food and drinks in school tuckshops
8. Creatively integrating healthy eating in the school curriculum
9. Make sure all children who need the National School Nutrition Programme even if they are not in schools zoned for the Programme access it
10. Expand National School Nutrition Programme to include healthy breakfast and healthy snacks such as fresh fruit and vegetables
11. Create penalties (using a demerit or other system) for students who purchase unhealthy food and drinks
12. Regulate what kinds of foods can be sold at school tuckshops, using restrictions decided by the Department of Basic Education or school governing body
13. Introduce national laws to increase the price of unhealthy foods and make healthy food cheaper
14. Training of School Governing Bodies on how to oversee school food providers ensure high food quality and safety, and how to work with health promoters and environmental health officers
15. Training of food providers and teachers about existing school nutrition guidelines and national policies such as the National Tuckshop Guidelines and the National School Nutrition Programme implementation guidelines
16. Train students to understand nutrition rules and report the breaking of those rules
17. Bring food vendors who sell healthy foods into the school community and invite them to special events to increase their commitment to the school nutrition rules
18. Stop the use of food as a reward in schools
19. Stop advertising of unhealthy food products to children, including promotional materials or billboards or signs in the school and surrounding areas
20. Educating parents about the nutrition rules and regulations controlling unhealthy foods at school
21. Formalise regular meetings between school staff and tuckshops about school nutrition

### Phase 3 results: Prioritisation of identified interventions

Thirty-eight participants completed the first survey round, of whom 31 (81%) participated in the second and 26 (68%) in the third survey round. Table 4 shows the response rate by stakeholder groups for each survey round, as well as the number of interventions.

**Table 4:** Response rate and number of interventions per Delphi rounds

	**Round 1 (%)**	**Round 2 (%)**	**Round 3 (%)**
Group 1 (Public primary school principals)	14 (36.8)	12 (38.7)	8 (30.7)
Group 2 (Public primary school nutrition coordinators and tuckshop managers)	9 (23.6)	5 (16.1)	5 (19.2)
Group 3 (Policy makers from the Department of Education and Health)	6 (15.7)	6 (19.3)	5 (19.2)
Group 4—Academia and civil society	9 (23.6)	8 (25.8)	8 (30.7)
Total number of stakeholders (*n* = number of interventions)	38 (*n* = 21)	31 (*n* = 11)	26 (*n* = 7)

In round 1, 11 of the 21 interventions passed the consensus agreement threshold to be included in round 2 (Table 5). Despite high consensus among stakeholders of the high importance of interventions 9 (ensuring access to the National School Nutrition Programme) and 13 (laws to influence pricing of foods), these were perceived as less feasible to implement compared to other interventions on the list. These interventions (9 and 13) sought to address structural barriers to a healthy environment. In round 2, the number of important and feasible interventions reduced to 7 for round 3. Although stakeholders agreed over the importance of all four interventions excluded after round 2 their feasibility was perceived to either be low, or the level of consensus agreement did not meet the inclusion criteria of QD ≤ 0.5.

**Table 5:** Stakeholders’ ranking of importance and feasibility of identified interventions in Delphi rounds

	**Round 1**		**Round 2**		**Round 3**
Intervention	Median Importance (QD)	Median Feasibility (QD)	Median Importance (QD)	Median Feasibility (QD)	Priority order (Borda count)
1	5 (0.5)	4 (1)			
2	3.5 (1.88)	3 (1)			
3	5 (0.5)	4 (0.5)	4 (0.5)	3.5 (0.625)	
4	5 (0.5)	4 (0.5)	4.5 (0.5)	4 (0.5)	6 (90)
5	5 (0.5)	5 (0.5)	5 (0.5)	4 (0.5)	2 (115)
6	5 (0.5)	4 (0.88)	5 (0.5)	4 (0.625)	3 (112)
7	5 (0.5)	5 (0.5)	5 (0.5)	4 (1)	
8	5 (0)	5 (0.5)	5 (0.5)	5 (0.5)	4 (104)
9	5 (0.5)	3 (0.88)			
10	5 (0.38)	4 (1.5)			
11	3 (1)	2 (0.5)			
12	5 (0.5)	4.5 (0.88)	5 (0.5)	4 (0.5)	1 (131)
13	5 (0.5)	3 (1)			
14	5 (0.5)	4 (1)			
15	5 (0.5)	4 (0.5)	5 (0.5)	4 (0.5)	5 (98)
16	4 (1)	4 (1)			
17	4 (0.88)	4 (0.5)	4 (1)	4 (1)	
18	3 (1)	3 (1)			
19	5 (0.5)	4 (0.88)	5 (0.5)	4 (1)	
20	5 (0.5)	4 (1)			
21	5 (0.5)	4.5 (0.88)	4 (0.5)	4 (0.5)	7 (72)

The result of round 3 ranking exercise is shown in Table 6. The relationship of these priority interventions to their theoretical underpinnings can be found in detail in Additional file 1. Interventions frequently related to existing policies (i.e., National School Tuckshop Guidelines) and available governance structures (i.e., the presence of school governing bodies). These were some of the few but key facilitators that stakeholders perceived as critical to leverage on when promoting healthier school food environments in Table 2. The priority interventions target the whole school including learners, tuckshops, staff, caregivers, and policy makers alike to create a school environment conducive to heathier diets. All seven interventions combined, stakeholder priorities targeted 18 of the 31 unique contextual drivers outlined in Table 2 that at the time of the study were perceived to limit the healthfulness of school food environments, with negative consequences on children’s nutrition.

**Table 6:** Interventions in ranked order of priority

	**Borda count**	**Intervention**
1	131	Regulate what kinds of foods can be sold at school tuckshops, using restrictions decided by the Department of Basic Education or School Governing Bodies
2	115	Train school staff through workshops and discussions to improve the nutrition environment within the school
3	112	Compulsory, child-friendly warning labels on all unhealthy food products
4	104	Creatively integrating healthy eating into the school curriculum
5	98	Training of food providers and teachers about existing school nutrition guidelines and national policies, such as the National Tuckshop Guidelines and the National School Nutrition Programme implementation guidelines
6	90	Introduce peer nutrition programmes, i.e., school gardens, science days, markets, and other programmes organised by the scholars
7	72	Formalise regular meetings between school staff and tuckshops about school nutrition

The intervention with the highest score (131) was a regulatory policy, and it sought to address 2 of the 31 contextual drivers in Table 2. These included the provisioning of cheap and unhealthy foods and beverages at school tuckshops and children’s and caregivers’ brand recognition of unhealthy drinks and foods.

The second intervention on the priority list fell within the environmental/social planning policy category. On the one hand, it aimed to address barriers related to the psychosocial capability of school staff who were perceived to lack awareness of existing guidelines on food and beverages. On the other, it sought to mitigate barriers related to the reflective motivation of school staff around the perceived difficulty in implementing school policies while sustaining their motivation to promote healthier dietary habits among caregivers and children.

The intervention that received the third highest priority score, child-friendly labels on unhealthy foods, was classified as legislation. It sought to enhance a key facilitator, which was awareness of the benefits of healthy eating and the harms of sugar, and aimed to address two key barriers outlined in Table 2. These included children’s and caregivers’ unhealthy eating habits/mindsets, including caregivers’ food provisioning; and children’s purchasing behaviour that was often guided by convenience.

## Discussion

Stakeholders in this study prioritised seven context-specific interventions to improve school food environments. These interventions had a high level of stakeholder buy-in and were perceived as feasible by those who would bear primary responsibility for their implementation – and thus could be a powerful starting point for the adoption of new or improved measures to strengthen school food environments. Prioritised interventions targeted a wide range of contextual factors influencing school stakeholders, policy makers, and children’s capability, motivation, and opportunity of having healthier foods within schools. While enhanced nutrition education was among the priorities, stakeholders did not believe that information provision alone was adequate to shift dietary practices within schools to healthier options. This is because understanding of the benefits of eating healthy and the harms resulting from unhealthy foods was seen as insufficient to facilitate healthier behaviours in the presence of other external barriers, such as affordability and availability of unhealthy foods within school premises. As such, prioritised interventions included rules that determine the quality of food sold and served in school, regulation of unhealthy food marketing, and nudging of healthy food behaviour such as labelling of unhealthy foods. Barriers to healthier school food environments also included more distal economic and structural realities, such as the importance of profit from the sale of unhealthy foods on local livelihoods and a lack of infrastructure in schools, for example, clean drinking water.

Prioritised interventions in this study could be regarded as a comprehensive package of interventions with buy-in from key stakeholders and future research could explore their combined implementation and effectiveness. Our findings also indicate that there is no need for new strategies. Existing food and nutrition policies can and should be leveraged in South Africa to improve school food environments. What is needed in these settings is to first adapt these existing interventions to respond to the context-specific nuances of a given environment. Second, in Gauteng primary schools, there is a need for strengthened governance mechanisms (including accountability and transparency) to enhance the implementation of existing policies, and strategies to minimise noncompliance and maximise enforcement. Furthermore, a supportive legal context will be necessary, where existing regulatory instruments (i.e., policies on food/nutrition, taxes, advertising, school health, education, and social protection) can be leveraged for mainstreaming the promotion of healthier school food environments. These features were reported to be critical for developing and implementing strong, effective, and mandatory policies for healthy school food provision and marketing restrictions in other LMICs [20].

Contextual factors identified during this study are not unique to South Africa but emerge as common influences on diet in other LMICs [20]. For example, limited resources in Mexican public schools led to reliance on unhealthy food industry sponsorship to fund school equipment like refrigerators [43]. Similarly, inadequate school funds in the Philippines resulted in having to fund a public school feeding scheme through sales of unhealthy foods which conflicted with introducing healthier food alternatives [44]. However, the nuances of which interventions are appropriate and feasible to address these challenges are context-specific. Our findings underscore recent calls in the literature for the need to generate local evidence and tailor global school nutrition policies to context [19, 20, 22]. This is important given the shortcomings we noted in the scope of policies included in the NOURISHING framework [37]. For example, by virtue of the development of policies in a high-income setting, the framework lacked interventions to respond to issues such as a lack of infrastructure or clean water, which are common LMIC challenges. The multifaceted context in which school nutrition policies are implemented requires a set of mutually reinforcing and complementary actions [22]. When interventions are delivered in a siloed approach, the potential benefit of even effective interventions might be hindered. For example, poor outcomes of a nutrition-education only intervention in Chile was attributed to the lack of accompanying changes in the school food environment [45].

Lastly, beyond the contextual factors identified in this study, recent evidence of policy implementation from South Africa and other LMICs provide invaluable lessons for anticipating and developing strategies to counteract possible food industry pushback. A review from 12 LMICs has reported how unhealthy food and beverage industry influence has limited the development and implementation of new or more stringent policies or regulations, including restricting sales of unhealthy foods and beverages in schools [46].

## Strengths and limitations

Qualitative and theory-based evidence, anchored in the lived experiences of key stakeholders has been lacking in the field of food environment research in LMICs. The strength and novelty of this study was its emphasis on and inclusion of diverse stakeholders, from schools, government, academia, and civil society, both in identifying what needs to change and informing the prioritisation of interventions. Nevertheless, caution should be adopted in interpreting the results as generalisable to other contexts. Findings represented the issues of public primary schools and priority interventions were designed to be specific to the context of urban settings in Gauteng. Contextual drivers of healthy school food environments and intervention priorities may differ in a rural setting due to a range of socioeconomic factors. Nevertheless, the robust research methodology used to identify context-specific issues and prioritise responsive interventions can be adapted and replicated in different settings, including peri-urban, semi-rural and rural areas. Additionally, the study did not collect data regarding the stakeholders’ age and years of professional experience, factors that could have influenced their selection of priority interventions. Nor did the study explore the views of students and caregivers except for those with a role in school tuckshop management and the school governing body. Addressing this gap could be the focus of future research where previously excluded groups could deliberate on the specific design of prioritised interventions. Such information could also be triangulated with past implementation experiences documented in the literature relevant to prioritised interventions. We report on some other logistical constraints in the study design paper [26].

## Conclusion

While the causes of unhealthy diet are broadly understood to emanate from the availability, affordability, quality, and acceptability of food in a given setting, this study demonstrates that the diets consumed are the result of a complex interplay of individual and structural factors. Moreover, the specific drivers within a given context have a nuance that ought to be considered when developing interventions to improve diet. This research provides an important overview of contextual drivers that influence the healthfulness of school food environments, and outlines priority actions that policy makers, school personnel, civil society and academia alike perceived as highly important and feasible to implement. Going forward, multisectoral action is required that enhance school stakeholders’ capabilities to utilise and benefit from existing healthy food guidelines within schools; motivation and opportunities to make healthy choice the easy choice.

## Supplementary Information

**Additional file 1.** Detailed overview of how evidence-based interventions were adapted and matched with contextual drivers through the application of the Behaviour Change Wheel.

## Data Availability

The dataset generated and analysed during the current study is not publicly available due to limitations of ethical approval with regards to participant confidentiality but are available from the corresponding author on reasonable request.

## Abbreviations

COM-B: Capability, Opportunity, Motivation and Behaviour
LMICs: Low- and middle-income countries
QD: Quartile deviation
TDF: Theoretical Domains Framework
QAS: Qualitative secondary analysis
