# Supplementary material for: Participatory prioritisation of interventions to improve primary school food environments in Gauteng, South Africa
Source: BMC Public Health. 2023 Jun 29;23:1263. doi: 10.1186/s12889-023-16101-z (PMC10308686; doi:10.1186/s12889-023-16101-z)
Supplement: Supplementary file 1 — Additional file 1. Detailed overview of how evidence-based interventions were adapted and matched with contextual drivers through the application of the Behaviour Change Wheel. [file 12889_2023_16101_MOESM1_ESM.docx]

| **Additional file 1 -** Detailed overview of how evidence-based interventions were adapted and matched with contextual drivers through the application of the Behaviour Change Wheel. | | | | | | | | |
| --- | --- | --- | --- | --- | --- | --- | --- | --- |
|  | **Contextual driver** | **COM-B Component** | **NOURISHING policy area** | **NOURISHING intervention** | **Behaviour Change Wheel Intervention Functions** | **Behaviour Change Wheel Policy Category** | **Context Specific Intervention** | **Plain language** |
|  | - Lack of school infrastructure to promote healthier eating (i.e., taps, covered eating space, fridges, gardens) (-) | Physical opportunity | Offer healthy foods and set standards in public institutions and other specific settings | Standards in social support programmes | Environmental restructuring | Environmental/ social planning, Service provision | Department of Basic Education increasing funding and resources to provide infrastructure such as water taps and nutrition gardens to schools | Government to provide resources to schools, for example taps, vegetable gardens and places for children to eat |
|  | - Cheap and unhealthy foods and beverages at school tuckshops (-) - Lack of affordable fresh nutritious foods in schools (-) - Difficulty in controlling vendors outside of school premises by school staff (-) - Fear of children getting sick from vendor foods (+/-) - School staff’s fear of negatively influencing vendors’ livelihoods (-) | Physical opportunity,  Reflective motivation,  Automatic motivations | Set incentives and rules to create a healthy retail and food service environment | Initiatives to increase the availability of healthier food in stores and food service outlets | Incentivization | Environmental/ social planning | Allow informal vendors selling healthier products to trade within school groups and recognize them as vendors and then vendors can be regulated | Recognise, regulate, and allow informal vendors to sell healthy food and drinks on school property |
|  | - Lack of affordable fresh nutritious foods in schools (-) - Influence of product characteristics (i.e., shelf life, pricing) on tuckshop stock (-) - Tuckshops’ fear of loss of profit if stocking healthy vs unhealthy foods (-) | Physical opportunity,  Automatic motivation | Use economic tools to address food affordability and purchase incentives | Targeted subsidies for healthy food | Enablement, Incentivization | Fiscal measures | Subsidies or tax exemptions for tuckshops selling healthy food | Incentivise school tuckshops to sell healthy food and drinks by giving subsidies or decrease tax |
|  | - Awareness of the benefits of healthy eating and the harms of sugar (+) - Perceived limited memory span of children to retain long-term health knowledge (-) - Perceived inability of younger children to control their health (-) - Difficulty of breaking children’ and caregivers’ unhealthy eating habits (-) - Children’s and caregivers’ individual preference for unhealthy over healthy foods (-) | Psychological capability,  Reflective motivation,  Automatic motivation | Give nutrition education and skills | Initiatives to train school children on growing food | Education, Training, Persuasion | Service provision, Environmental /social planning | Peer-to-peer nutrition programmes, experiential learning for children to understand the value and importance of healthy food through school gardens, science days, markets, and other programmes | Introduce peer nutrition programmes, for example school gardens, science days, markets, and other programmes organized by the scholars |
|  | - Difficulty of implementation of school policies (-) - School staff’s lack of awareness of guidelines on food and beverages (-) | Reflective motivation,  Psychological capability | Give nutrition education and skills | Training for teachers or those responsible for nutrition education of children | Persuasion | Environmental/ social planning | Consultations with staff to develop buy-in for interventions to improve school environment | Train school staff through workshops and discussions to improve nutrition environment within the school |
|  | - Awareness of the benefits of healthy eating and the harms of sugar (+) - Difficulty of breaking children’ and caregivers’ unhealthy eating habits/mindsets (-) - Convenience and affordability of unhealthy food guides caregivers’ food provisioning, and children’s purchasing (-) | Psychological capability,  Reflective motivation | Nutrition label standards and regulations on the use of claims and implied claims on food | Mandatory requirement that advertisements must carry a health message or warning | Persuasion | Legislation | Mandatory requirement on health messaging or warning on unhealthy food | Compulsory, child-friendly warning labels on all unhealthy food products |
|  | - Lack of affordable fresh nutritious foods in schools (-) - Convenience and affordability of unhealthy food guides caregivers’ food provisioning, and children’s purchasing (-) - Perceived inability of younger children to control their health (-) | Physical opportunity,  Automatic motivation,  Reflective motivation | Offer healthy foods and set standards in public institutions and other specific settings | Initiatives to increase the availability of healthier food in stores and food service outlets | Environmental restructuring, Enablement | Environmental/ social planning, Service provision | Increase the availability of healthier foods to enable children to make healthier purchases | Help children make healthier choices by increasing the availability and appeal of healthier food and drinks in school tuckshops |
|  | - Awareness of the benefits of healthy eating and the harms of sugar (+) - Perceived limited memory span of children to retain long-term health knowledge (-) | Psychological capability | Give nutrition education | Nutrition education on curricula | Education | Communication/ marketing, Service provision | Incorporating healthy eating across the curriculum in creative ways | Creatively integrating healthy eating in the school curriculum |
|  | - Mismatch of school location, children needing support and NSNP allocation policy (-) | Physical opportunity | Offer healthy foods and set standards in public institutions and other specific settings | Standards in social support programmes | Enablement | Environmental/ social planning | Audit and rework NSNP guidelines relating to the eligibility of schools and children to allow for less arbitrary inclusion criteria | Make sure all children who need the NSNP even if they are not in schools zoned for the Programme access it. |
|  | - Lack of affordable fresh nutritious foods in schools (-) - Convenience and affordability of unhealthy food guides caregivers’ food provisioning, and children’s purchasing (-) | Physical opportunity,  Reflective motivation | Offer healthy foods and set standards in public institutions and other specific settings | Fruit and vegetable initiatives in schools | Enablement, Incentivisation, Persuasion | Environmental/ social planning, Service provision | Increase availability of fruits and vegetables and provision of breakfast meals as part of the NSNP scheme | Expand NSNP to include healthy breakfast and healthy snacks such as fresh fruit and vegetable |
|  | - Fear of children getting sick from vendor foods (+/-) - Lack of adherence to food and beverage guidelines by children, school tuckshops, and vendors outside of schools (-) | Automatic motivation,  Psychological capability | Offer healthy food and set standards in public institutions and other specific settings | Mandatory standards for food available in schools including restrictions on unhealthy food | Coercion | Regulation | Create penalties for students purchasing from vendors | Create penalties (using a demerit or other system) for students who purchase unhealthy food and drinks |
|  | - Cheap and unhealthy foods and beverages at school tuckshop (-) - Children’s and caregivers’ brand recognition of unhealthy drinks and foods (-) | Physical opportunity,  Social opportunity | Offer healthy food and set standards in public institutions and other specific settings | Mandatory standards for food available in schools including restrictions on unhealthy food | Restriction, Environmental restructuring | Regulation | Possible restrictions introduced by the School Governing Bodies or Department of Basic Education on whether tuckshops can operate and what types of products can be sold at tuckshops | Regulate what kinds of foods can be sold at school tuckshops, using restrictions decided by the Department of Basic Education or School Governing Bodies |
|  | - Cheap and unhealthy foods and beverages at school tuckshop (-) - Tuckshops’ fear of loss of profit if stocking healthy vs unhealthy foods (-) | Physical opportunity,  Social opportunity,  Automatic motivation | Use economic tools to address food affordability and purchase incentives | Health-related food taxes | Restriction, Education | Fiscal measures | Fiscal policies to increase prices of unhealthy foods (and communication of such policies) | Introduce national laws to increase the price of unhealthy foods and make healthy food cheaper |
|  | - School staff’s and tuckshops’ feeling of pointlessness, discouragement due to lack of collective action among food outlets (-) - Difficulty of implementation of school policies (-) - Lack of adherence to food and beverage guidelines by children, school tuckshops, and vendors outside of schools (-) | Psychological capability,  Automatic motivation,  Reflective motivation | Give nutrition education and skills  Harness supply chain and actions across sectors to ensure coherence with health | (G) Training for teachers or those responsible for nutrition education of children;  (H) Governance structures for multi-sectoral/stakeholder engagement | Enablement | Environmental/ social planning, Guidelines | Training for School Governing Bodies to engage with food providers on food quality, including engaging with health promotors and environmental health officers | Training of School Governing Bodies on how to oversee school food providers ensure high food quality and safety, and how to work with health promoters and environmental health officers |
|  | - School staff’s lack of awareness of guidelines on food and beverages (-) - Lack of adherence to food and beverage guidelines by children, school tuckshops, and vendors outside of schools (-) | Psychological capability,  Social opportunity | Inform people about food and nutrition through public awareness  Give nutrition education and skills | (I) Development and communication of guidelines for specific food groups;  (G) Training for teachers or those responsible for nutrition education of children;  (G) Training for caterers and food service providers | Education, Training | Guidelines | Development, communication and training for food providers and teaching staff on existing school guidelines and national policies such as the National Tuckshop Guidelines and the NSNP implementation guidelines | Training of food providers and teachers about existing school nutrition guidelines and national policies such as the National Tuckshop Guidelines and the NSNP implementation guidelines |
|  | - Lack of adherence to food and beverage guidelines by children, school tuckshops, and vendors outside of schools (-) - Perceived limited memory span of children to retain long-term health knowledge (-) - Perceived inability of younger children to control their health (-) - Difficulty of breaking children’ and caregivers’ unhealthy eating habits (-) | Psychological capability,  Reflective motivation,  Social opportunity | Inform people about food and nutrition through public awareness | Development and communication of guidelines for specific food groups | Coercion, Modelling, Education, Training | Regulation | Introduce student reporting of policy breaches, including training on policy content | Train students to understand nutrition rules and report the breaking of those rules |
|  | - Difficulty of implementation of school policies (-) - Difficulty to control vendors outside of school premises (-) | Reflective motivation | Set incentives and rules to create a healthy retail and food service environment | Initiatives to increase the availability of healthier food in stores and food service outlets | Incentivisation | Regulation, Environmental/ social planning | Adoption of measures to incentivize compliance with policies such as welcoming food vendors to special events such as sports and cultural activities after hours. | Bring food vendors who sell healthy foods into the school community and invite them to special events to increase their commitment to the school nutrition rules |
|  | - School’s reward culture using unhealthy foods (-) - Children’s and caregivers’ individual preference for unhealthy over healthy foods (-) - Difficulty of breaking children’ and caregivers’ unhealthy eating habits (-) | Social opportunity,  Automatic motivation | Offer healthy food and set standards in public institutions and other specific settings | Mandatory standards for food available in schools including restrictions on unhealthy food | Restriction | Regulation | Restricting the use of foods as a reward in school settings and in tuckshops | Stop the use of food as a reward in schools |
|  | - Children’s and caregivers’ brand recognition of unhealthy drinks and foods (-) - Acknowledged support from donors (+/-) - Association of wealth status and unhealthy foods (-) | Physical opportunity,  Social opportunity, Automatic motivation | Restrict food advertising and other forms of commercial promotion | Mandatory regulation of food marketing in schools and more broadly | Persuasion, Environmental restructuring | Legislation | Introduce restrictions on child-directed marketing of unhealthy food products, including removing promotional materials related to unhealthy food | Stop advertising of unhealthy food products to children, including promotional materials or billboards or signs in the school and surrounding areas |
|  | - School staff’s intent to encourage healthier dietary habits among caregivers and children (+) - Difficulty of implementation of school policies (-) - Caregiver’s perception of school’s sole responsibility of ensure healthy diets for children (-) | Psychological capability,  Reflective motivation | Inform people about food and nutrition through public awareness | Development and communication of guidelines for specific food groups | Education | Guidelines, Regulation | Educating parents about the content of policies restricting unhealthy foods at school | Educating parents about the nutrition rules and regulations controlling unhealthy foods at school |
|  | - School staff’s and tuckshops’ feeling of pointlessness, discouragement due to lack of collective action among food outlets (-) - Difficulty of implementation of school policies (-) | Automatic motivation,  Reflective motivation | Harness supply chain and actions across sectors to ensure coherence with health | Governance structures for multi-sectoral/ stakeholder engagement | Enablement | Environmental/ social planning | Creation of coordination mechanisms between school staff and tuckshop staff | Formalise regular meetings between school staff and tuckshops about school nutrition |

( +) indicates a facilitator, (-) indicates a barrier; ( ±) indicates a barrier that can act as a facilitator. NSNP stands for National School Nutrition Programme.
